# A Meta-Analysis of the Efficacy of Prokinetic Agents against Glycemic Control

**DOI:** 10.1155/2019/3014973

**Published:** 2019-09-09

**Authors:** Yeon-Ji Kim, Woo Chul Chung, Seung Jae Lee

**Affiliations:** ^1^Department of Internal Medicine, The Catholic University of Korea, Seoul, Republic of Korea; ^2^Medical Library, The Catholic University of Korea, Seoul, Republic of Korea

## Abstract

**Background:**

Prokinetic agents are used in diabetic gastroparesis patients to improve gastric emptying and upper gastrointestinal (GI) symptoms. However, the efficacy of prokinetic agents against glycemic control is questionable. Therefore, we conducted a systemic review and meta-analysis to determine the efficacy of prokinetic agents against glycemic control.

**Methods:**

Randomized controlled trials (RCTs) evaluating the effect of prokinetics were identified by searching PubMed, Embase, and the Cochrane Library databases until April 2018. The primary outcome was changes in the mean value of glycosylated hemoglobin (HbA1c), fasting blood sugar (FBS), and fasting serum insulin (FINS). The pooled standardized mean differences (SMD) with 95% confidence intervals (CIs) were calculated by evaluating the strength of the association. We used the random effect models to analyze these markers. The effects of each component of the prokinetic agents on glycemic control were separately analyzed.

**Results:**

Five RCTs with 190 patients met the criteria and were included in the meta-analysis. There were statistically significant SMD between prokinetics and placebo-controlled groups with respect to the reduction of HbA1c (-1.141, 95% CI -1.843, -0.438; *P* < 0.01). No statistically significant differences were noted between the two groups for FBS (-1.270, 95% CI -2.613, -0.074; *P* = 0.06) and FINS (0.359, 95% CI -1.205~1.923; *P* = 0.65).

**Conclusions:**

Prokinetics have a positive effect on glycemic control. Further large-scale prospective studies are needed.

## 1. Introduction

Diabetic gastroparesis is a complication that often occurs in patients with long-standing diabetes, and it is characterized by chronic delayed gastric emptying without mechanical obstruction and upper GI symptoms. Diabetic gastroparesis occurs in about 25-55% of patients with diabetes [1]. The pathogenesis of diabetic gastroparesis has not been clearly defined, but it is known to be associated with autonomic neuropathy, enteric neuropathy, abnormalities of interstitial cells of Cajal and smooth muscle cells, acute hyperglycemia, and psychological dysfunction [2, 3]. The treatment aim of diabetic gastroparesis is to maintain an adequate glucose level, control upper GI symptoms, ensure adequate nutrition, improve gastric emptying, provide psychologic support, and prevent complications.

Prokinetic agents have been used in managing the symptoms of diabetic gastroparesis [4]. These include all compounds that have the pharmacological activity of modulating (stimulating or inhibiting) gastrointestinal motility. Motilin agonists, serotonin (5-hydroxytryptamine, 5-HT) receptor agonists, and dopamine antagonists have been mainly used for the treatment of GI diseases including diabetic gastroparesis. Several randomized control trials (RCTs) and experimental studies have shown the potential action of prokinetics as hyperglycemic inhibitors. However, it is not certain whether prokinetics are effective in glycemic control and if they help to modulate gastrointestinal motility. Therefore, we conducted an evidence-based review of the efficacy of prokinetics against glycemic control.

## 2. Materials and Methods

### 2.1. Search Strategy

The meta-analysis was conducted and reported in accordance with the Preferred Reporting Items for Systematic Reviews and Meta-Analyses (PRISMA) statement [5]. We searched RCTs published in English, which were comparative studies on the efficacy of prokinetic agents against glycemic control. PubMed, Embase, and Cochrane Library databases (undertaken by SJ Lee, Medical Library, the Catholic University of Korea, Seoul, Korea) were searched for RCTs until April 2018. To find specific RCTs, the following Medical Subject Headings (MeSH) terms and/or text words were used: Diabetes Mellitus, Type 2 OR Hyperglycemia OR Glucose AND Gastrointestinal Agents OR Metoclopramide OR Domperidone OR levosulpiride OR Cisapride OR mosapride OR tegaserod OR Erythromycin OR DA-9701.

### 2.2. Study Selection

Citations and abstracts of all retrieved studies were downloaded to Endnote X8.1 citation management software (Thomson Reuters, Philadelphia, PA, USA). After removing duplicated titles and abstracts, the retrieved articles were independently reviewed by two authors (Y.J. Kim and W.C. Chung). The full text of the relevant articles was checked against inclusion criteria and discrepancies, and any issues were resolved by consensus.

In the meta-analysis, the inclusion criteria were as follows: (1) RCTs, (2) studies on adult diabetic and prediabetic state patients, (3) glycemic control measured by HbA1c or FBS, (4) the control group received placebo for the same period as the treatment group, and (5) the treatment group received prokinetic agents for at least 1 week without any other GI medications such as gastric acid inhibitors or mucoprotective drugs. Studies were excluded if they were available only as an abstract, a review study, a case report, a study without raw data available for retrieval, a duplicate publication, a non-English publication, a crossover study design, or studies without a control group or data from a single experiment. Data without a mean value for the outcome between the two groups were also excluded.

### 2.3. Study Outcomes

The aim of this study was to analyze the effects of prokinetics compared to placebo on glycemic control defined by SMD of HbA1c and FBS. A fasting insulin level was also compared according to prokinetics usage. The effect of prokinetics on glycemic control according to the ingredients was additionally evaluated. Each drug component was categorized as a serotonergic agonist, dopamine antagonist, motilin agonist, and cholinergic agonist.

### 2.4. Statistical Analysis

The pooled SMD with 95% CIs was quantitatively evaluated. Heterogeneity among the studies was measured using Higgins' *I*^2^ statistics, with the value of >50% being indicative of statistical heterogeneity. When there was substantial heterogeneity (*I*^2^ > 50%), all analyses were based on the random effect model (DerSimonian-Laird method); otherwise (*I*^2^ < 50%), the fixed effect model (inverse variance method) was used. A meta-analysis of variance (ANOVA) was used for subgroup analysis based on each ingredient of the prokinetics. The analysis was performed when there were at least two related trials. All the above statistical analyses were conducted using the R language meta-package ver. 3.4.3 (R Foundation for Statistical Computing, Vienna, Austria).

## 3. Results

### 3.1. Literature Search

**Figure 1:**
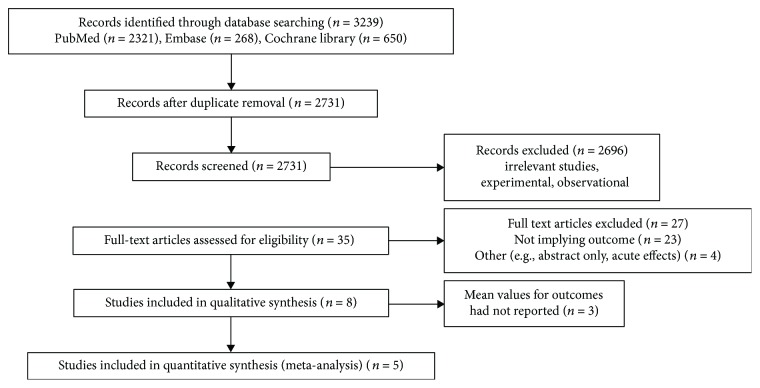
Flow diagram of the study.

The process of literature search and study selection is shown in Figure 1. A total of 3,239 published articles were retrieved from searching the three databases, MEDLINE, Embase, and the Cochrane Library. Among them, 508 were duplicates and 2,696 were excluded based on the title and abstracts. After full-text reviewing, five articles were considered for the meta-analysis. Three studies were not included because the required values such as mean and standard deviation (SD) were not reported [6–8].

### 3.2. Study Characteristics

The characteristics of the eligible studies are summarized in Table 1. In the five RCTs, 190 participants were identified (prokinetics, 106; placebo, 84). Two trials used a motilin agonist (erythromycin), two trials used a serotonergic agonist (mosapride), and one study used a dopamine antagonist (levosulpiride). Treatment duration ranged from 2 to 24 weeks, and the duration of diabetes ranged from 6.8 to 23 years. Three RCTs included participants with type 2 diabetes, one study included type 1 diabetic (IDDM) patients, and the other included patients with impaired glucose tolerance (IGT) (Table 2) [9–13]. All prokinetics were orally administered. Two RCTs (Melga JP, 1997 and Ueno N, 2001) showed improvement in bowel movements after taking prokinetic agents, but the evaluation method for gastric emptying time was heterogeneous and difficult to perform [9, 12].

### 3.3. Comparative Efficacy of Prokinetics against Glycemic Control

**Figure 2:**
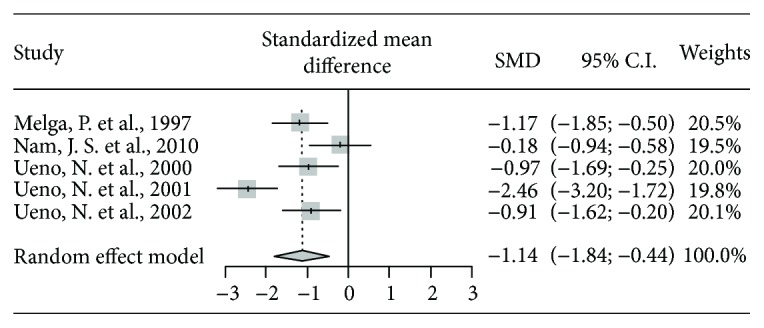
The results of the meta-analysis for HbA1C.

The effect of prokinetics on HbA1c was examined, and four of five studies reported a statistically significant improvement in HbA1c between the prokinetics and control groups (Table 3 and Figure 2). Prokinetics led to a decrease in HbA1c by 1.141. (Higgins' *I*^2^ = 79.0, 95% CI: -1.843, -0.438, *P* < 0.01). In the subgroup analysis based on ingredients, the motilin agonist group (subgroup 2: -1.714, 95% CI: -3.177, -0.251, *P* = 0.02) showed better efficacy than the 5-HT agonist group (subgroup 1: -0.560, 95% CI: -1.090, -0.052, *P* = 0.12) with a significant difference.

**Figure 3:**
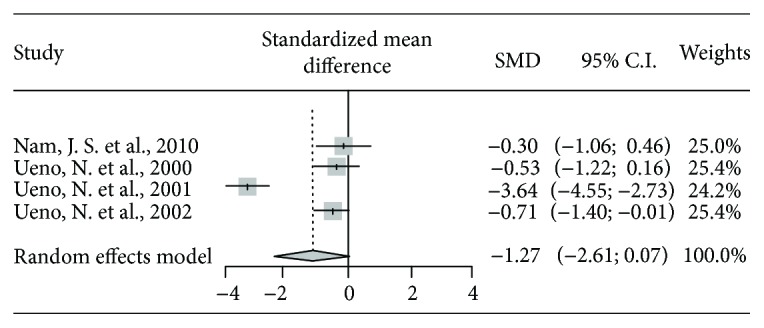
The results of the meta-analysis for FBS.

Four studies reported results on FBS by comparing the prokinetics group with placebo (Table 4 and Figure 3). However, there was a nonsignificant reduction in FBS with the use of prokinetics (-1.270, Higgins' *I*^2^ = 92.0%, 95% CI: -2.613, 0.074, *P* = 0.06).

### 3.4. Comparative Efficacy of Prokinetics on FINS

**Figure 4:**
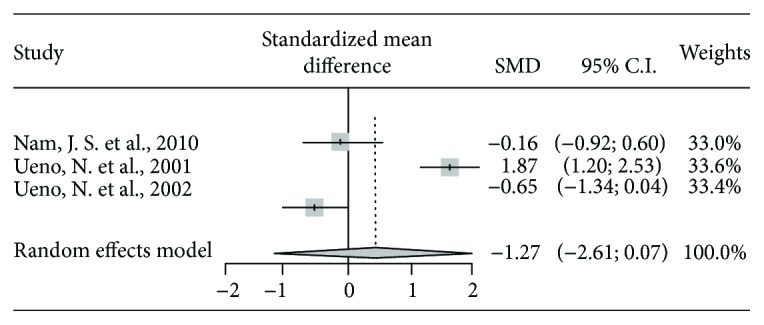
The results of the meta-analysis for fasting insulin.

The effect of prokinetic agents on FINS was examined in three studies. Two studies (Nam et al. [10], Ueno et al. [13]) showed lower FINS in the prokinetics group compared to placebo, while one study showed higher FINS in the prokinetics group. The meta-analysis indicated that there was no statistically significant change in FINS (*P* = 0.06) (Table 5 and Figure 4).

## 4. Discussion

The gut plays a crucial role in glucose homeostasis by aiding digestion, absorption, and assimilation of ingested nutrients [14]. The determinants of postprandial and preprandial glycemic levels are associated with meal composition, gastric emptying, insulin secretion, small intestinal glucose absorption, and hepatic and peripheral glucose metabolism [15].

The mechanisms by which prokinetics improve glucose metabolism are unclear, although several hypotheses have been proposed, such as improvements in insulin sensitivity or much greater secretion of plasma insulin to increase intraduodenal glucose loads. Some animal studies showed that erythromycin and motilin stimulate GI motility and the cyclic release of insulin and pancreatic polypeptides from the pancreas through the vagal-cholinergic muscarinic pathway [16, 17]. In addition, serotonin (5-HT) is a neurotransmitter that has been implicated in the regulation of diverse physiological processes, including cell growth and differentiation, neuronal development, and the regulation of blood glucose concentrations [18]. Few studies showed the potential role of 5-HT receptors in insulin secretion and action [13, 19]. Cisapride, a 5-HT_4_ agonist with partial 5-HT_3_ antagonism, might have a stimulatory effect on the endocrine pancreas [20]. However, a recent study suggests that the specific 5-HT_4_ receptor agonist, mosapride, decreases plasma glucose concentration without stimulating insulin secretion. It was presumed that it may improve insulin sensitivity by increasing serotonergic activity [10].

Gastric emptying of solid food is known to consist of two phases: the lag phase (the meal is transported from the fundus to the antrum) and the postlag phase (the solid food particles are propelled through the pylorus) [21]. Glycemic values influence gastric emptying and limit glycemic fluctuation. Hyperglycemia slows gastric emptying by prolonging the lag phase and decreasing the postlag emptying rate, whereas hypoglycemia accelerates it [22]. Reduced proximal gastric tone, suppression of antral pressure waves, and stimulation of pyloric contractions are possible mechanisms of this. The relationship between gastric emptying and plasma glucose control is bidirectional, which involves nutrient absorption and hormonal effect [23].

The Diabetes Control and Complications Trial (DCCT)/Epidemiology of Diabetes Interventions and Complications (EDIC) study showed that delayed gastric emptying was associated with early and long-term hyperglycemia [24, 25]. Therefore, delayed gastric emptying was considered a manifestation of autonomic neuropathy, resulting in poorly controlled diabetes. There are relatively few studies and limited evidence on the effects of hyperglycemia on delayed GI motility [26]. A recent study revealed a significant difference between postprandial glucose and continuous glucose throughout the day [27]. It was shown that the glucose values from continuous glucose monitoring were associated with delayed gastric emptying unlike postprandial glucose.

Gastric emptying involves a complex interplay among the GI smooth muscle, gastric pacemaker cell networks, the so-called interstitial cells of Cajal, and neurohormonal systems, particularly inhibitory feedback arising from the interaction of nutrients with the small intestine [28]. The small intestine plays a major role in glucose homeostasis and in duodenal gastric feedback including vagovagal reflex and action of glucagon-like peptide-1 (GLP-1), peptide YY (PYY), and cholecystokinin (CCK) from the distal small intestine involved in regulating gastric emptying. These hormones stimulate insulin, suppress glucagon, and potentially reduce energy intake [29, 30].

Despite controversies, several current studies showed that an acceleration of the gastric emptying occurs following obesity surgery and more antral resections can lead to faster gastric emptying time [31–35]. In addition, some studies showed the efficacy of bariatric surgery against diabetes mellitus (DM) remission by accelerating meal transit after bariatric surgery [36–38]. Nutrient sensing, which occurs mainly in the proximal jejunum, would be stimulated by undigested food delivered into the distal jejunum after bariatric surgery. It affects the reduction of hepatic glucose production. In addition, when the entire jejunum is bypassed, the secretion of insulin resistance factor might be inhibited with consequent normalization of insulin sensitivity [39]. Recent endoscopic procedures such as gastrojejunal sleeve placement and jejunal mucosal ablation therapy improved glycemic indexes by bypassing the small intestine and improving malabsorption. Prokinetics might change gastric and intestinal emptying by changing the bowel movements, which may lead to rapid nutrient migration such as after bariatric surgery. It also can improve glycemic control via better synchronization between the onset action of exogenous insulin and the release of nutrients in the intestine and absorption by promoting gastric emptying.

This systemic review on the efficacy of prokinetics on glycemic control suggests that prokinetics reduced the glycemic marker, HbA1c, with a significant reduction in HbA1c implying long-term fluctuations in blood glucose concentration. However, prokinetics had no stimulatory effect on insulin secretion. This indicated that prokinetics have significant effects in reducing glucose metabolism in diabetic and prediabetic patients, and this action suggests that a mechanism other than insulin secretion is involved.

Our study showed a decrease in HbA1c with prokinetics use, which is consistent with a decrease in overall glucose and not with fasting glucose. There is a subtle difference in the assessment of the metabolic status of blood glucose as a consequence of FBS and HbA1c used in the diagnosis of diabetes. Fasting blood glucose involves evaluation of the state of glucose that remains stable in the body after a temporary increase in exogenously injected glucose is resolved, whereas HbA1c reflects the mean blood glucose level not just at the time of sampling but immediately before the test because it increases with the concentration and time of glucose in contact with hemoglobin. The FBS test is limited by low sensitivity and relatively large fluctuations [40]. Therefore, the decrease in HbA1c in this study may be a more meaningful result of overall blood glucose reduction rather than the FBS level. We believe that the results of this analysis should be interpreted carefully considering that the *P* value was not obtained statistically because of the small sample size and the limitations of FBS itself.

Although the studies in this meta-analysis excluded one-time studies, there were few studies that used long-term medication. Given the nature of the chronic disease, long-term research will likely need to demonstrate the benefits of prokinetics on glycemic control. Generally, prokinetics have few side effects associated with long-term maintenance. However, careful consideration is needed in choosing prokinetics. Attention should be paid to the CNS effect of levosulpiride and metoclopramide and the association between long-term oral erythromycin and poor tolerance, modest efficacy, and the development of tachyphylaxis. Prokinetics added to diabetic medication are expected to be noninvasive compared to newly designed invasive methods for the treatment of the metabolic syndrome. In particular, the dual effects of chronic dyspepsia and glycemic control are to be expected and it is also remarkable in terms of cost-effectiveness.

There are some limitations to consider when interpreting our findings. First, the studies included in this analysis had small sample sizes. In addition, the number of studies for subgroup analysis according to the ingredients of the prokinetics was also limited. Second, we could not directly assess the change in gastric or intestinal emptying by prokinetics because of the difference in the test methods used in each study. The effects of gastrointestinal motility may be different in each subgroup of prokinetics, and this result may be related to glucose levels, but it was difficult to compare this directly in this meta-analysis. Third, the treatment period in all studies was relatively short, such as 2-8 weeks, except one study with erythromycin. The glycemic marker, HbA1c, may determine the average blood glucose levels within the previous three weeks. Comparisons of HbA1c in the absence of an adequate treatment period may have the potential to underestimate the values.

## 5. Conclusions

The meta-analysis shows that prokinetics may be effective in significantly improving both gastroparesis symptoms and glycemic control. A further well-designed large-scale prospective study should be performed to determine the long-term effect of prokinetics on glycemic control.

## Figures and Tables

**Table 1:** Main characteristics.

Studies	Treatment	Mechanism of action	Treatment period
Melga, P. et al., 1997	Levosulpiride	Dopamine antagonist	24 weeks
Nam, J. S. et al., 2010	Mosapride	5-HT_4_ agonist	2 weeks
Ueno, N. et al., 2000	Erythromycin	Motilin agonist	4 weeks
Ueno, N. et al., 2001	Erythromycin	Motilin agonist	4 weeks
Ueno, N. et al., 2002	Mosapride	5-HT_4_ agonist	8 weeks

5-HT_4_: 5-hydroxytryptamine receptor 4.

**Table 2:** Baseline characteristics.

Studies	Age (years)		Sex (male/female)		BMI (kg/m^2^)		Duration of diabetes (years)	
	T	C	T	C	T	C	T	C
Melga, P. et al., 1997^∗^	45 ± 2	43 ± 2	8/12	9/11	21 ± 1	22 ± 1	23 ± 2	21 ± 2
Nam, J. S. et al., 2010^†^	50 ± 11	48 ± 9	14/6	8/2	25.3 ± 1.7	25.8 ± 1.2	—	—
Ueno, N. et al., 2000^∗^	55.0 ± 3.0	56.0 ± 2.0	14/5	8/7	23.2 ± 0.6	23.4 ± 0.8	7.0 ± 1.0	6.0 ± 1.0
Ueno, N. et al., 2001^†^	55.5 ± 2.4	54.0 ± 2.8	12/18	10/12	24.5 ± 1.2	24.7 ± 1.2	8.0 ± 1.0	7.0 ± 1.0
Ueno, N. et al., 2002^∗^	60 ± 4	59 ± 3	7/10	8/9	26.4 ± 1.1	25.8 ± 1.0	7.0 ± 2.3	6.8 ± 1.4

^∗^Mean ± SE for continuous variables; ^†^mean ± SD for continuous variables. BMI: body mass index; T: treatment; C: control.

**Table 3:** The results of meta-analysis for HbA1c.

Studies	Treatment			Control			Standardized mean diff.	95% lower CI	95% upper CI	Weights	*P* value
	*N*	Mean	SD	*N*	Mean	SD					
Melga, P. et al., 1997	20	5.7	0.95	20	6.8	0.89	-1.171	-1.848	-0.495	20.5%	
Nam, J. S. et al., 2010	20	5.4	0.5	10	5.55	1.2	-0.184	-0.944	0.577	19.5%	
Ueno, N. et al., 2000	19	7.6	0.87	15	8.6	1.16	-0.969	-1.689	-0.249	20.0%	
Ueno, N. et al., 2001	30	7.8	0.2	22	8.3	0.2	-2.462	-3.200	-1.724	19.8%	
Ueno, N. et al., 2002	17	7.67	0.78	17	8.5	0.99	-0.909	-1.620	-0.199	20.1%	
Total (random effect model)							-1.141	-1.843	-0.438		<0.01
Subgroup 1 (Nam, J. S. et al., 2010; Ueno, N. et al., 2002) (random effect model)							-0.560	-1.090	-0.052		0.12
Subgroup 2 (Ueno, N. et al. 2000; Ueno, N. et al., 2001) (random effect model)							-1.714	-3.177	-0.251		0.02

*P* value of the test of heterogeneity among studies = 0.0008; Higgins' *I*^2^ = 79.0% (49.9%, 91.2%).

**Table 4:** The results of meta-analysis for fasting blood glucose.

Studies	Treatment			Control			Standardized mean diff.	95% lower CI	95% upper CI	Weights	*P* value
	*N*	Mean	SD	*N*	Mean	SD					
Nam, J. S. et al., 2010	20	6	0.9	10	6.3	1.1	-0.301	-1.065	0.462	25.0%	
Ueno, N. et al., 2000	19	9.7	2.06	15	11.1	3.1	-0.532	-1.223	0.158	25.4%	
Ueno, N. et al., 2001	30	8.2	0.9	22	11	0.5	-3.368	-4.549	-2.276	24.2%	
Ueno, N. et al., 2002	17	8.51	1.4	17	9.6	1.61	-0.705	-1.401	-0.010	25.4%	
Total (random effect model)							-1.270	-2.613	0.074		0.06
Subgroup 1 (Nam, J. S. et al., 2010; Ueno, N. et al., 2002) (random effect model)							-0.522	-1.036	-0.010		0.04
Subgroup 2 (Ueno, N. et al., 2000; Ueno, N. et al., 2001) (random effect model)							-2.070	-5.113	0.973		0.18

*P* value of the test of heterogeneity among studies < 0.0001; Higgins' *I*^2^ = 92.0% (82.8%, 96.3%).

**Table 5:** The results of meta-analysis for insulin.

Studies	Treatment			Control			Standardized mean diff.	95% lower CI	95% upper CI	Weights	*P* value
	*N*	Mean	SD	*N*	Mean	SD					
Nam, J. S. et al., 2010	20	34.51	20.09	10	38.03	25.11	-0.157	-0.917	0.603	33.0%	
Ueno, N. et al., 2001	30	42	5.8	22	31.9	4.6	1.867	1.202	2.532	33.6%	
Ueno, N. et al., 2002	17	40.8	22.68	17	54.7	19.05	-0.648	-1.340	0.044	33.4%	
Total (random effect model)							0.359	-1.205	1.923		0.65
Subgroup 1 (Nam, J. S. et al., 2010; Ueno, N. et al., 2002) (random effect model)							-0.426	-0.937	0.086		0.10

*P* value of the test of heterogeneity among studies < 0.0001; Higgins' *I*^2^ = 93.2% (83.6%, 97.2%).
